# The More the Tubular: Dynamic Bundling of Actin Filaments for Membrane Tube Formation

**DOI:** 10.1371/journal.pcbi.1004982

**Published:** 2016-07-06

**Authors:** Julian Weichsel, Phillip L. Geissler

**Affiliations:** Department of Chemistry, University of California, Berkeley, California, United States of America; McGill University, CANADA

## Abstract

Tubular protrusions are a common feature of living cells, arising from polymerization of stiff protein filaments against a comparably soft membrane. Although this process involves many accessory proteins in cells, in vitro experiments indicate that similar tube-like structures can emerge without them, through spontaneous bundling of filaments mediated by the membrane. Using theory and simulation of physical models, we have elaborated how nonequilibrium fluctuations in growth kinetics and membrane shape can yield such protrusions. Enabled by a new grand canonical Monte Carlo method for membrane simulation, our work reveals a cascade of dynamical transitions from individually polymerizing filaments to highly cooperatively growing bundles as a dynamical bottleneck to tube formation. Filament network organization as well as adhesion points to the membrane, which bias filament bending and constrain membrane height fluctuations, screen the effective attractive interactions between filaments, significantly delaying bundling and tube formation.

## Author Summary

The necessary biophysical conditions for the formation of tubular membrane protrusions by polymerizing actin filament bundles have not yet been fully understood. For this reason we introduce a novel grand canonical simulation model that describes stochastic polymerization of filaments against a fluctuating fluid membrane, while only considering a minimum set of biological proteins. Although still relatively simple and highly tractable, our model explicitly accounts for thermal fluctuations of membrane and filaments, stochastic and quantized polymerization dynamics at the filament tip, cooperativity of multiple filaments, and steric interactions between all model constituents in a physically realistic way. This approach enables us to go well beyond previous static zero-temperature theoretical considerations to filament bundling and explore the physical origins of membrane tube formation dynamics on length and time scales that are currently inaccessible to both experiments and atomistically detailed simulations. Our results suggest a membrane mediated dynamical transition from single filaments to cooperatively growing bundles as an important dynamical bottleneck to tubular protrusion.

## Introduction

**Fig 1:**
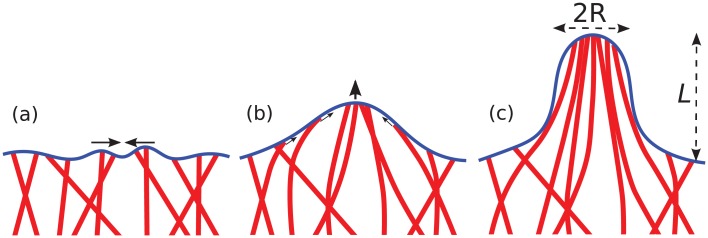
Snapshots in time from (a) to (c), illustrating schematically the spontaneous bundling of actin filaments. Interaction of filaments (red) with a biological membrane (blue) dynamically organizes a collection of individually polymerizing filaments into a dense aligned bundle in which polymerization becomes highly cooperative. This mechanism yields actin-driven formation of a membrane tube, with height *L* and radius *R*, requiring no additional accessory proteins.

Individual cells generate tubular membrane protrusions in order to sense and interact with their environment [1]. The necessary work for their formation is performed by the directed polymerization of a tightly aligned parallel actin filament bundle against the load of the cell membrane [2]. Although the core of the underlying molecular machinery required for actin driven membrane tube formation is well known, a key role in the process has been attributed to different accessory proteins in different experimental scenarios [3]. Recently, in-vitro reconstituted branched actin networks, containing only a minimum set of three purified proteins (i.e. actin, Arp2/3, and N-WASP) and growing from outside against the membrane of a giant unilamellar vesicle, were shown to yield filopodia-like protrusions [4]. This finding highlighted the importance of subtle physical interactions between a reduced set of molecular ingredients in bundling filaments and forming membrane tubes. It suggests a much less elaborate mechanism for bundling and protrusion, principally involving effective attractive interactions between neighboring filaments that are mediated by nearby small-amplitude deformations of the membrane due to the individual filaments’ stochastic polymerization (cf. Fig 1(a)). When this bundling process eventually accumulates a sufficient number of filaments to overcome membrane resistance, filopodia-like structures emerge.

In recent years, several studies explored the nature of filopodia protrusion. Specifically the impact of the stochastic nature of capping and polymerization reactions [5–7], of actin network crosslinkers and reorganization [8–10], and of mechanical (in-)stability [11] on filopodia formation and growth have been analyzed. Here, we develop a novel grand canonical membrane simulation method to establish a comprehensive statistical dynamics framework for such phenomena. By relaxing specific rather limiting model assumptions that were made for simplification in previous theoretical approaches [4, 12–15], we arrive at a physically highly plausible, yet relatively simple, computational model system. In detail, our model explicitly accounts for thermal fluctuations of membrane and filaments, stochastic and quantized polymerization dynamics at the filament tip, cooperativity of multiple filaments, and steric interactions between all model constituents. Within this framework we are able to reproduce previously established results for the force-extension curves of membrane tubes [16] and the growth rate of a single filament against an obstacle [12, 17]. Much more importantly, our simulation method allows a detailed analysis of the dynamics of the tubulation process in filament-driven membrane protrusion. It had been shown before, by computing static zero-temperature minimum elastic energy shapes of two nearby membrane deformations as a function of their distance, that the membrane can induce an effective lateral attraction between the two [4, 14]. Within our dynamical approach, we find that filament tips that are attracted to a remote primary membrane protrusion are initially arrested in a metastable state in which net polymerization ceases. A rare combination of bending and polymerization fluctuations is necessary to overcome the energy barrier to joining the protruding bundle. The typical escape time from this arrested state strongly depends on subtle polymer network parameters, like filament length and orientation, as well as possible adhesion points to the membrane, which constrain height fluctuations. As the waiting time for this bundling process competes with other network related processes like filament capping and network turnover, it is expected to be an important parameter in the biological system. By analyzing many individual trajectories of semiflexible filaments polymerizing against a fluctuating membrane patch, we establish a comprehensive statistical understanding of the dynamical transition from independently growing individual filaments to cooperatively polymerizing filament bundles that efficiently drive membrane tube extension.

## Results

### Grand canonical membrane simulation model

In typical in-vitro experiments, e.g. in [4, 18], micrometer-length membrane tubes protrude from the surface of a large vesicle, which provides a fixed reference frame supporting the deformation. The vesicle’s membrane also serves as a reservoir of lipid molecules, from which the tube draws material as it elongates. Because a tube constitutes a very small fraction of the total lipid population, its area can change many-fold without influencing the surface tension imposed by the much larger lipid reservoir.

In order to avoid the great computational expense of representing the lipid reservoir in numerical simulations explicitly, we extended a widely used dynamically triangulated surface model [19, 20] to allow for fluctuations in lipid population within a grand canonical ensemble, where surface tension is held fixed. Specifically, we examine a periodically replicated square membrane patch, fluctuating around its flat ground state according to Metropolis-Hastings grand canonical Monte Carlo (GCMC) dynamics. We account for the membrane energy with a discretized form of the standard Helfrich Hamiltonian [21–23],

$$H=\int \text{dS}\left[\frac{\kappa }{2}{\left(2H\right)}^{2}+\gamma \right]\;,$$ (1)

with mean curvature *H*, bending rigidity *κ*, and surface tension *γ*. Fluctuations in lipid population are achieved by Monte Carlo moves that attempt to change the number of vertices in the triangulated surface. Appropriate acceptance criteria for these moves are derived in S1 Text. Addition and removal of membrane area is regulated by a fugacity *z* characterizing the implicit lipid reservoir. For the case of an incompressible fluid membrane, *z* can be related directly to the membrane tension *γ*,

$$z=C\exp\left[-\frac{\gamma }{\rho k_{B}T}\right]\;,$$ (2)

where *ρ* is the constant lateral density of the fluid surface and *k*_B_*T* is the thermal energy scale. Other system specific parameters are combined into the constant *C* (see S1 Text for details).

**Fig 2:**
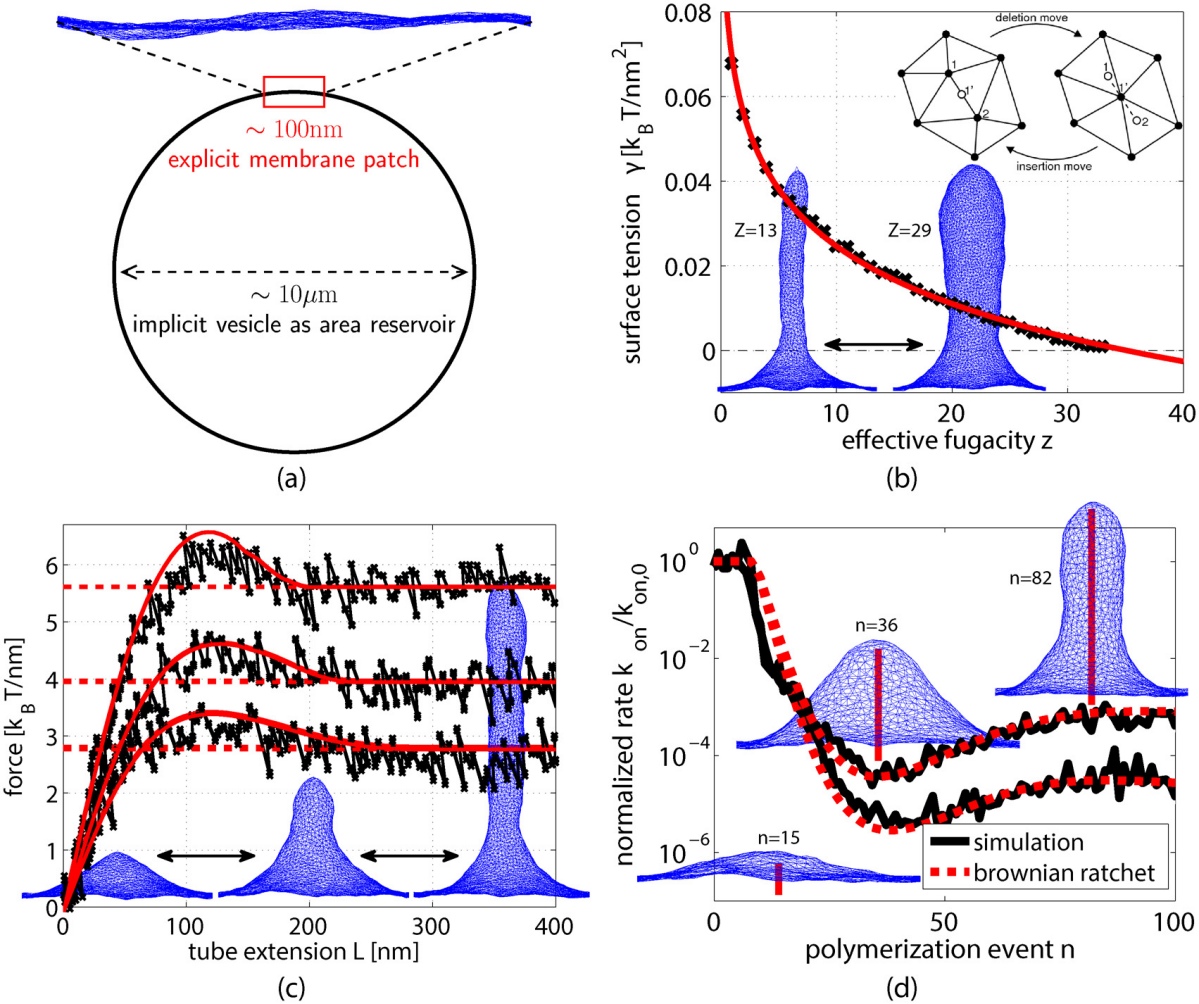
GCMC simulation of a triangulated membrane patch. (a) Sketch of the simulation setup. The area of a small fluctuating membrane patch is coupled grand canonically to an implicit lipid reservoir at constant surface tension, mimicking the large excess of vesicle area in typical experiments. (b) The control parameter fugacity *z* can be mapped to surface tension *γ* in simulation (black crosses) and fitted using Eq (2) (red line). Simulation snapshots are shown for fugacities, *z* = 13 and *z* = 29. Inset sketch: GCMC node removal and insertion moves. (c) Membrane force-extension curves (black) compared to zero-temperature calculations at *κ* = 20*k*_B_*T* and *γ* = {0.005;0.01;0.02}*k*_B_*T*nm^−2^ (red solid lines from bottom to top, respectively). Asymptotic pulling force, $f_{0}=2\pi \sqrt{2\kappa \gamma }$ (red dashed lines). Simulation snapshots are shown at tube extensions *L* around 72nm, 188nm, and 540nm, with *γ* = 0.01*k*_B_*T*nm^−2^. (d) Effective polymerization rate *k*_on_(*n*)/*k*_on,0_ (black solid) of a single rigid filament comprising *n* monomers compared to the expected near-equilibrium behavior (red dashed), for *κ* = 20*k*_B_*T* and *γ* = {0.005;0.01}*k*_B_*T*nm^−2^ (upper and lower curves, respectively). Snapshots are shown for *γ* = 0.01*k*_B_*T*nm^−2^.

A membrane patch simulated in this way can be manipulated as if it were part of a much larger vesicle (Fig 2(a)). For instance, membrane tubes can be pulled from the initially flat patch by applying an additional external potential to the triangulated surface (Fig 2(b) and 2(c)), much as in optical tweezer experiments [18]. The equilibrium radii of such tubes are simply determined (at zero temperature) by the membrane’s rigidity and tension, $R_{0}=\sqrt{\kappa /(2\gamma )}$ [16]. We exploit this relationship to determine values of the constants in Eq (2) for various *z* (Fig 2(b)). As a quantitative test for our simulation methodology, we calculated force-extension relations *f*(*L*) for membrane tube formation from GCMC sampling. As shown in Fig 2(c), our results are consistent with numerical zero-temperature calculations [24]: The computed pulling force initially increases with extension *L*, then decreases towards an asymptotic plateau value, $f_{0}=2\pi \sqrt{2\kappa \gamma }$, in the limit of long tubes [16].

As a further test of our methods, we examined the irreversible polymerization kinetics of a single rigid filament (i.e., depolymerization rate *k*_off_ = 0, persistence length *L*_p_ → ∞) growing against a simulated membrane patch. The position and orientation of the filament’s base is held fixed throughout the simulation. Stochastic polymerization attempts, occurring at rate *k*_on,0_ and resulting in an increase *δ*_fil_ = 2.7nm in filament length, were accepted whenever permitted by constraints of excluded volume, i.e., whenever the membrane’s fluctuating shape could accommodate monomer addition (see S1 Text and Materials and Methods Sec. A for additional details of the simulations). The normalized rate of successful polymerization, *k*_on_(*n*)/*k*_on,0_, decays rapidly with the polymerization event number *n* in membrane simulations (see Fig 2(d)). This is consistent with the decrease as expected from near-equilibrium theory, *k*_on_(*n*)/*k*_on,0_ ≃ exp[−(*f*(*L*)*δ*_fil_)/(*k*_B_*T*)], with retraction force *f*(*L*) of the membrane at extension *L* [12, 25]. Under typical in-vitro conditions (i.e. globular actin concentration ∼ 10*μ*M and nonzero depolymerization rate *k*_off_), net polymerization of a single actin filament ceases early in protrusion once the steady state condition *k*_on_(*n*)/*k*_on,0_ = *k*_off_/*k*_on,0_ ≃ 10^−2^ is met. Forming extended membrane tubes thus typically requires cooperative polymerization of multiple neighboring filaments, which share the load of the membrane.

### Cooperative polymerization leads to membrane tubes

**Fig 3:**
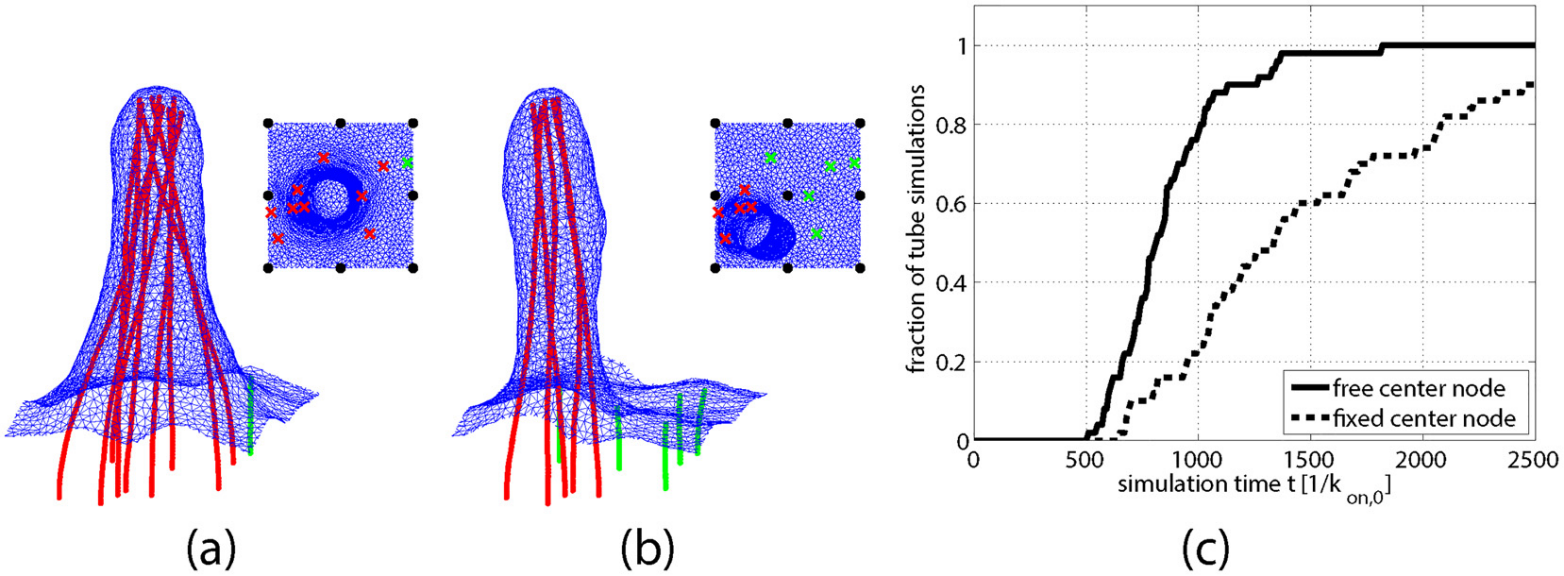
Immobile membrane nodes screen membrane mediated attractions between filaments. (a) & (b) Two simulated tubes at equal height, generated by simulations with the same initial filament conditions and parameters, differing only by the presence in (b) of one additional immobilized membrane node at the center. Bundle formation is delayed and fewer filaments join the bundle in (b) compared to (a) (cf. S1 & S2 Movies). Insets: Top view of the simulation snapshot indicating immobile nodes (black circles) and initial positions of tube filaments (red crosses) and as yet unbundled filaments (green crosses). (c) Fraction of 50 simulation trajectories with and without the central frozen node that yield a membrane tube before time *t*, plotted as a function of *t*. At any given time, fewer of the constrained trajectories (dashed line) formed tubes than in the unconstrained case (solid line).

To establish the necessary physical conditions for cooperative filament growth, we analyzed 50 independent trajectories of collections of *N*_fil_ = 10 growing, structurally fluctuating (persistence length *L*_p_ = 15*μm*), and additionally depolymerizing (*k*_off_/*k*_on,0_ = 10^−2^) filaments anchored at uniformly random lateral positions below the membrane. To fix the membrane’s base relative to the filaments’ anchor points, we constrain the positions of three boundary nodes of the membrane to be constant throughout the simulation runs. The inset of Fig 3(a) shows in black the location of these *frozen* nodes (together with their periodically replicated copies) within the membrane patch.

Despite the lack of additional accessory proteins for filament bundling and crosslinking in our model, all simulated trajectories eventually yielded tight cooperative filament bundles and subsequently a single polymerization-driven membrane tube of at least 260nm in height. For sufficiently large filament density, bundle formation is thus only a matter of time. It is initiated by the interaction of individual filaments with the membrane, which leads to bending and directed growth towards a single point of protrusion as sketched in Fig 1(b). Roughly half of the tubes formed before a simulation time of 800/*k*_on,0_, while the slowest tube formation process required about 1800/*k*_on,0_ to reach this height (cf. solid line in Fig 3(c)). This range in tube formation times reflects a distribution of waiting times for filament bundling by the membrane.

### Membrane induces dynamic filament bundling

**Fig 4:**
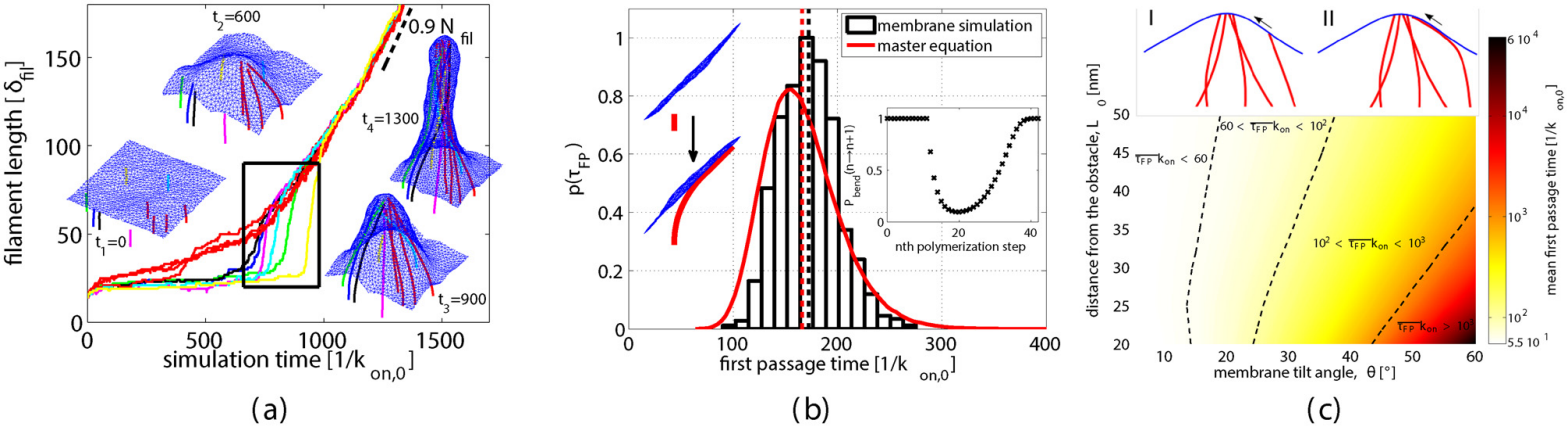
Membrane induced dynamic filament bundling. (a) Contour lengths of growing filaments vs. simulation time. *N*_fil_ = 10 semiflexible filaments (*L*_p_ = 15*μ*m) are initialized at random positions 50nm below and perpendicular to the membrane. Initially, a collection of nearby filaments (red) begin to bundle where filament density is high. Remote filaments (colors other than red) then bend and subsequently join the bundle in a cascade of dynamic transitions (black box). Insets: Snapshots at intermediate simulation times (cf. S3 Movie). (b) Left inset: An individual filament with a fixed base at *L*_0_ = 60nm below the membrane and at relative angle *θ* = 45° grows against the membrane. Rare polymerization events lead to a bent filament state growing tangentially to the membrane (cf. S4 Movie). Main figure: First passage time distribution *p*(*τ*_FP_) to reach the bent and growing filament state. GCMC simulation results (black bars) are compared to the master equation Eq (5) (red solid line). Vertical dashed lines indicate mean first passage times (MFPT). Right inset: Probability *P*_bend_(*n* → *n* + 1) that a gap of sufficient size is available to add a monomer to a filament of contour length *nδ*_fil_. (c) Heat map of the MFPT for the filament bending transition vs. the initial angle and distance of the filament relative to the membrane. Color scale is logarithmic, and cooler colors indicate longer waiting times. Top inset: Each individual dynamic filament bending transition I→II can be understood as a single filament interacting with a predeformed membrane.

In simulation, the accumulation of filaments into a tight bundle occurs through a cascade of dynamical transitions. In the early stages of a representative trajectory Fig 4(a), significant net polymerization and membrane protrusion take place only where the lateral density of filaments happens to be high (red in Fig 4(a)). The filaments’ growing (“barbed”) ends are pushed together by forces from the membrane, as described in [4, 14]. For growth conditions we have studied, the resulting premature bundle is not sufficient to overcome the membrane’s restoring force. Net polymerization therefore stalls, and the interfacial deformation fluctuates around a steady state height.

The recruitment of additional filaments to the premature bundle occurs sequentially, as each one traverses a dynamical bottleneck evidenced by sudden changes in its growth rate (Fig 4(a), colors other than red). Remote filaments experience a membrane-induced attraction to the bundle, but it is offset by the elastic cost of their own bending. Their tendency to join the bundle is thus not a direct consequence of mechanical forces on the barbed end. It is instead a product of the nonequilibrium nature of these dynamics (which generate net filament growth), together with the inevitability of fluctuations that create sufficient space for polymerization and the bias provided by the bundle’s deformation of the membrane.

Once the bundle amasses enough filaments to generate polymerization forces greater than *f*_0_, it grows steadily at a rate that increases with each additional recruitment event. These elongation kinetics suggest a high degree of cooperativity, with nearly equal sharing of the membrane’s load. Perfect load sharing, in which each of *N* filaments grows against a force *f*_0_/*N*, would yield a net polymerization rate *k*_on_ for each filament:

$$\frac{k_{\text{on}}}{k_{\text{on},0}}≃\exp\left[-\frac{f_{0}\delta_{\text{fil}}}{Nk_{B}T}\right]-\frac{k_{\text{off}}}{k_{\text{on},0}}\;.$$ (3)

In simulations we observe bundle elongation rates of nearly this magnitude, with an effective value of *N* that is about 90% of the true bundled filament population. The empirical effective polymerization rate of a complete bundle (i.e., Eq (3) with *N* set to 0.9*N*_fil_) is indicated by the black dashed line in Fig 4(a). Based on the measured radius, the membrane tension of the fully grown tube, *γ* = *κ*/(2*R*^2^), is consistent with the corresponding tube pulling simulation in Fig 2(b) at the same fugacity *z* = 21, i.e. *γ* ≃ 0.01*k*_B_*T*nm^−2^.

To analyze in detail the kinetics of recruiting filaments to a premature bundle, we focus on the simplified scenario sketched in Fig 4(c). Here, the bundle’s influence on a distant tagged filament is represented by a static average tilt angle *θ* and the vertical distance *L*_0_ between the filament anchor point and the membrane due to the bundle’s protrusion (cf. S1 Text, Fig S3 for a sketch indicating these parameter definitions). Growing beneath such a tilted patch of membrane, the tagged filament adds monomers at rate *k*_on,0_—*k*_off_ until approaching the membrane within a distance *δ*_fil_. Further polymerization awaits a sufficient fluctuation in the shape of the membrane and/or the filament. The most likely of these fluctuations involve bending of the filament toward the bundle (cf. inset sketch Fig 4(c)). Monomer addition locks in the resulting filament curvature, reducing the cost of further filament deformation and thus enhancing subsequent growth. Once the ground-state filament orientation at its barbed end is parallel to the tilted membrane, polymerization is again unimpeded and very rapid. Through this facile growth, the tagged filament quickly joins the nascent bundle.

First passage time statistics for reaching the bent state can be understood thoroughly in the context of a simplified model for growth kinetics. As equilibration of membrane and filament shape fluctuations is fast relative to the timescale of polymerization, we assume that structural fluctuations which create sufficient space for monomer addition occur with probabilities corresponding to thermal equilibrium, independent of growth history. The rate *k*_on_(*n*) for polymerization of a tagged filament already comprising *n* monomers is then given by

$$k_{\text{on}}\left(n\right)=k_{\text{on},0}P_{\text{bend}}\left(n\to n+1\right)\;,$$ (4)

where *P*_bend_(*n* → *n* + 1) is the equilibrium probability of a filament bending fluctuation that creates a gap of sufficient size *δ*_fil_ between membrane and filament. The S1 Text presents an analytical approximation for *P*_bend_(*n* → *n* + 1) that considers only the softest modes of deforming the membrane and filament. This result, which we use below, is plotted in Fig 4(b) inset.

The set *k*_on_(*n*) of effective polymerization rates informs a master equation for stochastic growth dynamics,

$$\begin{array}{ccc} \partial_{t}P\left(n,t\right) & = & k_{\text{off}}\left[P(n+1,t)-P(n,t)\right]\\ & & +k_{\text{on}}\left(n-1\right)P\left(n-1,t\right)\\ & & -k_{\text{on}}\left(n\right)P\left(n,t\right)\;, \end{array}$$ (5)

where *P*(*n*, *t*) denotes the probability that the tagged filament is composed of exactly *n* monomers at time *t*. We computed probability distributions *p*(*τ*_FP_) of the first passage time by numerically solving Eq (5). The results of this approximate treatment, plotted in Fig 4(b), agree well with detailed simulations of a single fluctuating filament growing against a membrane that fluctuates about a uniformly tilted state (see S1 Text details and the S4 Movie of the simulation).

The average waiting time 〈*τ*_FP_〉 to reach the bundle can be directly calculated from Eq (5) [26]. As shown in Fig 4(c), it varies strongly with the membrane tilt angle and filament length. Modest changes in these two key parameters alter the mean first passage time by orders of magnitude. Hence, our results suggest that the efficiency of dynamic bundling and, as a direct consequence, the rate of membrane tube formation is highly sensitive to even subtle structural changes in the underlying actin network architecture.

To test the impact of suppressed membrane height fluctuations on protrusion formation, we performed 50 additional simulations, using identical filament base positions as before, but with an additional frozen membrane node at the center of the patch (cf. Fig 3(a) and 3(b) insets). In a laboratory context, these constrained vertices could represent transient binding of filaments within a growing network to membrane-bound nucleation promoting factor N-WASP [27]. Due to this additional constraint on the membrane, the waiting time for filament bundle formation indeed increases substantially. Furthermore, the mature tubes that do form include fewer filaments in the growing bundle. The filaments that fail to join the bundle in this case are separated by an immobile node from the primary protrusion, as illustrated by representative simulation snapshots in Fig 3(a) and 3(b) (cf. S1 and S2 Movies of these simulations). This depletion of the bundle reduces its effective polymerization force, strongly delaying the emergence of protrusions and slowing their subsequent growth.

### Compatible filament orientations contribute in protrusion

The simulations and analysis described so far considered all filaments in the actin network to be growing in the same direction, normal to the initial plane of the membrane. Real networks, however, include filaments with a substantial range of orientations. Previous work has shown that this diversity has important consequences for reconstituted actin networks and migrating cells [28, 29]. Given the sensitivity of filament recruitment kinetics to the angle between filament and membrane, we expect orientational diversity within a network to also significantly impact dynamics of bundling and protrusion.

To explore the roles of filament alignment in tube formation, we advanced growth trajectories from initial conditions in which filament orientations were assigned randomly. In detail, we selected each filament’s initial polar angle from a Gaussian distribution centered at zero (with the polar axis pointing along the membrane’s normal vector) and with standard deviation std; its azimuth was selected uniformly without bias. We then monitor which filaments join a tube bundle at various stages of its development, as assessed by the tube’s height *L*. Different trajectories require different amounts of time to achieve the same value of *L*. The time at which the bundling transition occurs also varies from trajectory to trajectory, but the corresponding value of *L* is consistent. We performed 50 independent simulations for the case std = 20°, and another 50 for the case std = 40°.

**Fig 5:**
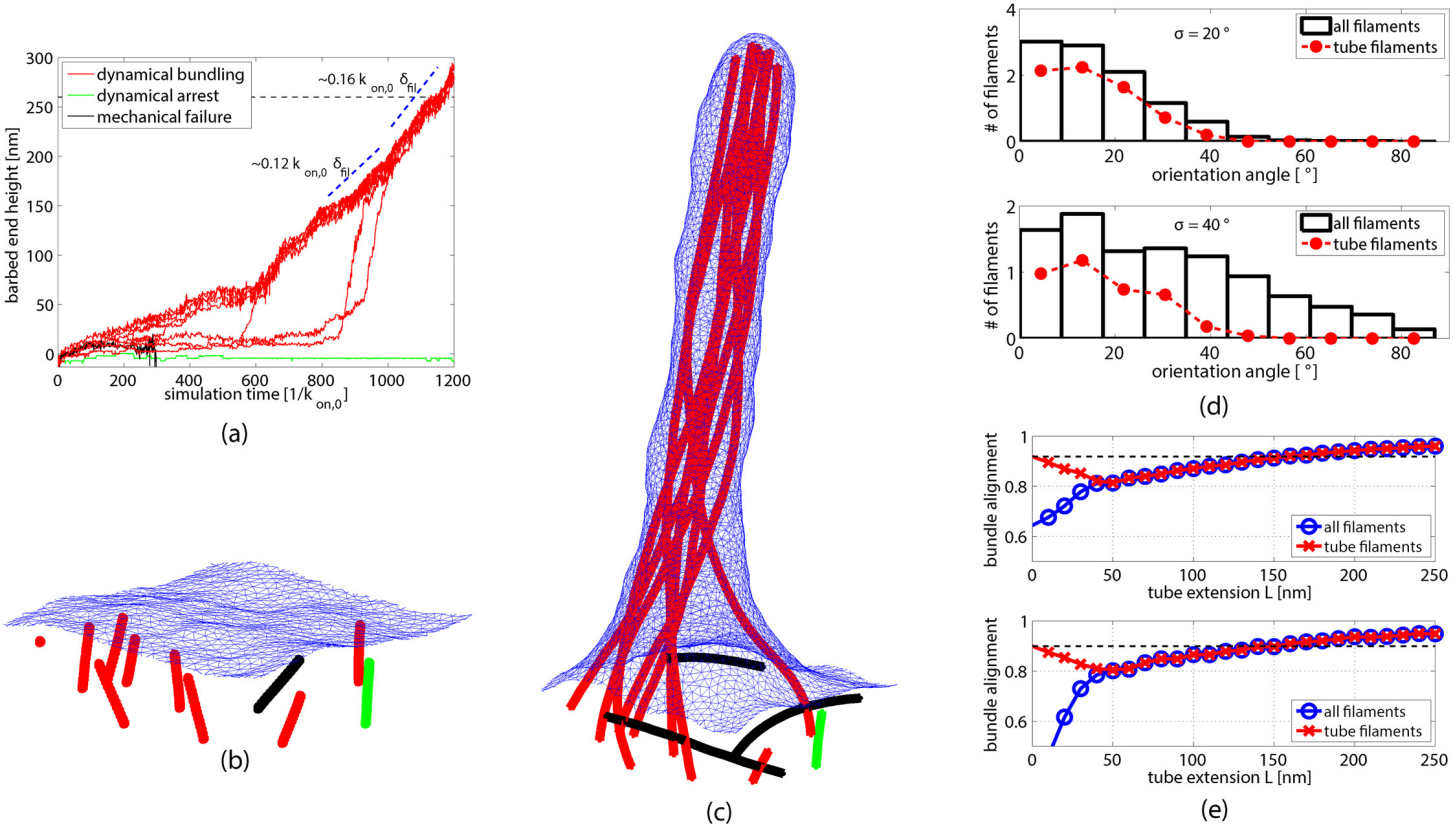
Filament orientation in bundles organized by interactions with a fluctuating membrane. (a) Polymerization of *N*_fil_ = 10 filaments whose base orientations are distributed with standard deviation std = 20° in the polar angle, shown for a single representative trajectory. At the time (black dashed horizontal line) that a tube of significant height (260nm) has formed, filaments can be classified into three distinct groups: (i) filaments that merged into the tube bundle (red), (ii) unbundled filaments that remain unbent (green), and (iii) filaments that mechanically failed, bent away from the membrane, and do not further contribute in protrusion (black). (b) & (c) Snapshots from the same tube-forming simulation considered in (a), taken at times before (b) and after (c) a membrane tube has formed (cf. S5 Movie). Filaments are colored according to the same classification scheme as in (a). (d) Distribution of filaments’ base orientations in two sets of 50 simulations, one with std = 20° (upper figure) and the other with std = 40° (lower figure). Black bars show statistics for all filaments, while the red dashed lines include only those filaments that belong to a tube of height *L* ≥ 260nm after a time 2500/*k*_on,0_. (e) Average alignment of filaments’ barbed ends, plotted as a function of tube height *L* for the same simulations as in (d). Blue curves include all filaments in this average, while red curves include exclusively filaments that are part of the tube-forming bundle. Horizontal black dashed lines show the values of these red curves at *L* = 0 for comparison.

The sequence of events in these trajectories is very similar to that previously described for filaments that all point in the same direction. A cascade of filament bundling events again accumulates filaments into a bundle (colored red in Fig 5) to form the tube. And all filaments that join the bundle subsequently contribute in load sharing and accelerate tube protrusion. The principal distinction is that some poorly aligned filaments fail to join the tube even at very long times.

A well-developed tube (*L* = 260 nm) typically includes filaments whose orientations lie within ∼ 40° of the surface normal. Filaments initially pointing outside this cone have either stalled at this stage (still awaiting recruitment to the bundle) or have failed mechanically and grown away from the membrane (as indicated by green and black coloring in Fig 5(a)–5(c)). For std = 20° this discrimination excludes only a small fraction of filaments. Many more filaments fail to join the mature bundle in the case std = 40°. Among filaments included in the tube, the distribution of initial orientations is very similar for the two values of std (see Fig 5(d)). Robust tube formation thus primarily requires a sufficient density of filaments at orientations that are compatible with bundling.

In addition to selecting filaments with sufficiently aligned initial orientations, bundling and protrusion significantly bias filaments’ orientation at the growing barbed end. Fig 5(e) shows the degree of alignment among barbed end orientations for filaments that eventually join the bundle (see Materials and Methods Sec. A for details), plotted as a function of tube height. These barbed ends are highly aligned already at low tube extension, reflecting the orientation selection process discussed above. At small membrane deformation, 0 ≤ *L* ≤ 50nm (i.e. before a major filament bundle has formed), alignment of these filaments transiently declines due to dynamic bending in a direction tangential to the membrane and subsequent bundling. Strong barbed end alignment is restored within the mature membrane tube. In long tubes, ≥ 150nm, the degree of alignment even exceeds its initial value, which is indicated by the black dashed horizontal line in Fig 5(e). Once filaments are recruited to protrude the membrane tube, they are corralled into a highly parallel bundle that efficiently drives tube extension.

## Discussion

In this work, we introduced a novel grand canonical simulation method for a fluctuating biological fluid membrane, based on a randomly triangulated surface, within a statistical ensemble of constant surface tension. Coupling the system to an implicit lipid reservoir, while at the same time explicitly accounting for thermal fluctuations and excluded volume constraints, our computational method is very well suited to efficiently simulate typical experimental vesicle assays and, more generally, biological processes in which the relevant membrane area changes over time.

By additionally including quantized and stochastic polymerization kinetics of fluctuating filaments, we identified and quantified key necessary microscopic conditions for filament polymerization-driven membrane tubes, which are extremely difficult to access experimentally. Our simulations revealed a cascade of single filament bending transitions as an important dynamical bottleneck to filament bundling and subsequent tubular protrusion formation. Even subtle changes in structural filament network parameters, such as filament orientation and length or additional constraints on the membrane’s height fluctuations have a profound impact on the waiting time for filament bundling. Inside the cell, this waiting time competes not only with the timescale of other polymer network related processes, like capping and actin turnover, but also with the viscous response time of the treadmilling actin gel. In this biological context, the waiting time for filament bundling will directly impact the rate of membrane tube formation and filopodia emergence in biological systems, or might even prevent their occurrence altogether.

These results suggest a possible explanation for unexpected experimental observations in reconstituted assays of actin-driven membrane tube formation [4]. Specifically, the number of filopodia-like tubes on vesicles was found to decline upon increasing Arp2/3 or N-WASP concentration, despite the concomitant increase in filament density expected by the biochemical perturbation. While higher filament density should enhance the collective force of polymerization, the denser filament network likely also features more numerous adhesions to the membrane. In our analysis, the resulting suppression of height fluctuations could indeed strongly diminish filament bundling and subsequent formation of linear protrusions.

Our model could be straightforwardly extended to account for the mechanics and dynamics of an explicitly crosslinked actin network, and of various actin associated proteins. Other parameter regimes of the model will also be worthwhile to explore. At even larger actin filament density, packing effects and the resulting thickness of the tubular actin bundle will become important as has been highlighted before [30–32]. Our results could be directly tested in biochemical experiments by manipulating the filament orientation distribution of the polymerizing actin network [33] or by incorporating artificial adhesion points into a reconstituted membrane. Together with such extensions and related measurements, our approach promises to enable a thorough microscopic understanding of the combined biochemical and biophysical requirements for the formation of actin-driven membrane protrusions.

## Materials and Methods

### Filament-membrane simulation

Each of the grand canonical Monte Carlo simulations feature a square membrane patch with linear size in the range of 200–300nm. The size of the membrane patch mostly determines the cutoff for long wavelength fluctuations. In the biological system we expect such long wavelength fluctuations to be efficiently damped by tethers between the membrane and the relatively rigid growing actin network. In simulations we are choosing the patch size to be able to resolve the smallest important wavelength on the size of the membrane’s thickness. To constrain the center of mass motion of the membrane patch vertically, three equidistant boundary nodes (and their periodic images) were immobilized (except where explicitly stated differently in the main text). Fluctuating actin filaments are included into the Metropolis MC scheme as worm-like chains, using the standard discretized Hamiltonian in combination with free rotation and crankshaft MC moves. The base segment of each discretized filament is fixed in position and orientation throughout the entire simulation. The membrane and filament MC quasi-dynamics can be mapped to physical dynamics for small step size. In this spirit, we related the timescale of filament and membrane MC dynamics by comparing the equilibration time of relevant normal modes for the process under consideration (see S1 Text for details). Additionally, in the dynamical simulation we account explicitly for stochastic, quantized (de-)polymerization events in which filaments change their contour length by 2.7nm with a kinetic MC approach that draws a random reaction time and filament that is subject to the corresponding reaction [34]. The effects of volume exclusion due to the membrane (with nodes assigned a diameter of 5nm) and other filaments (node diameter 10nm) on these biochemical kinetics are also included explicitly. Unless otherwise noted in the main text, we used typical in-vitro conditions for actin on- and off-rates, *k*_on,0_/*k*_off_ = 10^2^.

### Zero-temperature membrane calculations

Minimum energy shapes of the membrane at zero-temperature were calculated using Surface Evolver [24].

### Filament alignment measure

Filament segment alignment *a* in Fig 5(e) was measured by averaging barbed end orientations for filaments that eventually joined the mature bundle, $a\left(L\right)=|\sum_{i=1}^{N}{\hat{o}}_{i}|/N$, where ${\hat{o}}_{i}$ are the unit orientation vectors of filament segments whose height lies between *L* and *L* + Δ*L*, with Δ*L* = 10nm. Averages included results from all simulations that eventually formed a tube of minimum height, 260nm, before a given maximum time, 2500/*k*_on,0_.

## Supporting Information

S1 Text Supporting information text. (PDF)

S1 Movie Simulation of filament ensemble growth against a freely fluctuating membrane (cf. Fig 3(a)). (MP4)

S2 Movie Simulation of filament ensemble growth against a fluctuating membrane with one additional immobilized node (cf. Fig 3(b)). (MP4)

S3 Movie Filament-driven tube formation simulation (cf. Fig 4(a)). (MP4)

S4 Movie Single filament bending transition under consideration in Fig 4(b). (MP4)

S5 Movie Filament-driven tube formation simulation corresponding to Fig 5(a)–5(c). (MP4)
