# Supplementary material for: The More the Tubular: Dynamic Bundling of Actin Filaments for Membrane Tube Formation
Source: PLoS Comput Biol. 2016 Jul 6;12(7):e1004982. doi: 10.1371/journal.pcbi.1004982 (PMC4934920; doi:10.1371/journal.pcbi.1004982)
Supplement: S1 Text — (PDF) [file pcbi.1004982.s001.pdf]

The more the tubular: dynamic bundling of actin filaments for membrane tube formation – Supporting Information

Julian Weichsel[1,*] and Phillip L. Geissler[1,†]

[1]*Department of Chemistry, University of California, Berkeley, CA 94720, United States*

I. GRAND CANONICAL FILAMENT-MEMBRANE SIMULATION MODEL

In order to simulate a fluctuating fluid membrane at constant surface tension, we extended a widely used dynamically triangulated surface model [1] with an implicit grand canonical particle reservoir.

A. Acceptance criteria for grand canonical Monte Carlo moves

In order to preserve detailed balance along the grand canonical Monte Carlo moves, here we are deriving acceptance criteria for the proposed vertex-splitting (creation move) and -merging (deletion move). Considering the transition between the two microstates ν and ν', which hold $N+1$ and N membrane nodes respectively (cf. Fig. S1), detailed balance is obeyed if,

$$\frac{\text{acc}(\nu \to \nu')}{\text{acc}(\nu' \to \nu)} = \frac{\text{gen}(\nu' \to \nu)}{\text{gen}(\nu \to \nu')} \frac{P_{\nu'}}{P_\nu} , \tag{S1}$$

where $\text{acc}(\nu \to \nu')$ is the probability of accepting a transition from state ν to state ν', which is generated with probability $\text{gen}(\nu \to \nu')$. The relative Boltzmann weight of the two states ν' and ν is,

$$\frac{P_{\nu'}}{P_\nu} = \Lambda^3 \exp\left[-\frac{E_{\nu'} - E_\nu}{k_\text{B}T} + \frac{\gamma}{\rho k_\text{B} T}\right] , \tag{S2}$$

with thermal de-Broglie wavelength Λ, membrane surface tension γ, and internal vertex density of the triangulation ρ.

*Electronic address: julian.weichsel@bioquant.uni-heidelberg.de

†Electronic address: geissler@berkeley.edu

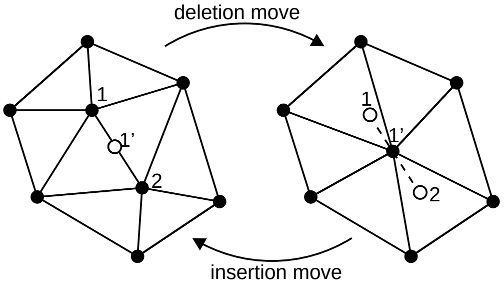

FIG. S1: Illustration of the grand canonical Monte Carlo move for the insertion/deletion of nodes into a local neighborhood of the triangulated surface.

To propose the trial move $\nu \to \nu'$, we draw a random linker of the triangulation and merge the two connected nodes at positions $\mathbf{r}_N$ and $\mathbf{r}_{N+1}$ to the position $\mathbf{r}'_N$ at their center of mass. For generating the reverse transition, a random membrane node is drawn at $\mathbf{r}'_N$ and split in two nodes at random positions $\mathbf{r}_N$ and $\mathbf{r}_{N+1}$ located at opposing positions on a sphere shell with center $\mathbf{r}'_N$ and random diameter between the hard-sphere membrane node diameter d_{node} and the maximum linker length $l_{\max}$. Subsequently, one of the possible ways to triangulate the involved neighborhood to the new pair of nodes is selected uniformly randomly. This procedure corresponds to generation probabilities,

$$\text{gen}(\nu \to \nu') = \frac{\delta(\mathbf{r}'_N - \frac{1}{2}(\mathbf{r}_N + \mathbf{r}_{N+1}))}{L_\nu} \tag{S3}$$

$$\text{gen}(\nu' \to \nu) = \frac{\int\limits_{d_{\text{node}}}^{l_{\max}} \int\limits_0^{\pi} \int\limits_0^{2\pi} p_l p_\phi p_\theta \delta(\mathbf{r}_N - (\mathbf{r}'_{N+1} - \hat{\mathbf{r}}\frac{l}{2}))\delta(\mathbf{r}_N - (\mathbf{r}'_N + \hat{\mathbf{r}}\frac{l}{2}))\text{d}l\text{d}\phi\text{d}\theta}{N_{\nu'} T_{\nu'}}, \tag{S4}$$

including the total number of membrane linkers L_ν in state ν, the total number of membrane nodes $N_{\nu'}$ in state ν', and all possible triangulations for the given neighborhood $T_{\nu'}$ in state ν' . For the creation move ($\nu' \to \nu$), the linker length l between the two split nodes as well as polar and azimuth angle of the linker orientation are drawn randomly according to the probability density distributions p_l, p_ϕ, and p_θ respectively. For feasible acceptance rates, we chose $p_l = \frac{1}{l_{\max} - d_{\text{node}}}$, $p_\theta = \frac{1}{2\pi}$, and $p_\phi = \frac{1}{2}\sin(\phi)$, where the latter is realized by inverse transform sampling, i.e. $\phi = \arccos(2u - 1)$ [9]. This ultimately leads to the acceptance criteria,

$$\text{acc}(\nu \to \nu') = \min\left\{1, \frac{d_{\text{node}}^2 L_\nu}{z\, l^2 T_{\nu'} N_{\nu'}} \exp\left[-\frac{E_{\nu'} - E_\nu}{k_{\text{B}}T}\right]\right\} \tag{S5}$$

$$\text{acc}(\nu' \to \nu) = \min\left\{1, \frac{z\, l^2 T_{\nu'} N_{\nu'}}{d_{\text{node}}^2 L_\nu} \exp\left[+\frac{E_{\nu'} - E_\nu}{k_{\text{B}}T}\right]\right\}, \tag{S6}$$

with $N_\nu = N_{\nu'} + 1$ and $L_\nu = L_{\nu'} + 3$ and our new parameter of the simulation, controlling tension, the effective fugacity,

$$z = \frac{4\pi \left(l_{\mathrm{max}} - d_{\mathrm{node}}\right) d_{\mathrm{node}}^2}{\Lambda^3} \exp\left[-\frac{\gamma}{\rho k_{\mathrm{B}} T}\right] . \tag{S7}$$

B. Gauging time in Monte Carlo simulations

1. Timescale for the relaxation of structural fluctuations

It has been shown previously, that Monte Carlo algorithms can be used to approximate overdamped physical dynamics [2]. In order to simulate the active dynamic process of actin filament polymerization against the fluctuating membrane, we are introducing an effective time scale to the combined membrane-filament MC simulation. By monitoring the relaxation time of a relevant normal mode of the structural equilibrium fluctuations for membrane and filament and comparing to their expected theoretical values, we are able to map the simulation time in terms of MC sweeps to physical units. For the sake of simplicity we are considering a flat membrane in free fluid. While this is well justified at this point to estimate the order of magnitude of the timescale of relaxations, it is known that a cylindrical membrane tube can give rise to different dynamics [3].

Fig. S2 is showing the decay in autocorrelation of structural fluctuations of the membrane model (a) and a worm-like chain (WLC) simulation (b). From the Helfrich Hamiltonian in Monge gauge and a WLC Hamiltonian in similar approximation, we obtain a theoretical estimate for the decorrelation time of each normal mode amplitude ($h_{\mathbf{q}}$ and a_q respectively),

$$\left\langle \left(h_{\mathbf{q}}(t+\Delta t) - h_{\mathbf{q}}(t)\right)^2 \right\rangle = \frac{2 A k_{\mathrm{B}} T}{\kappa q^4 + \gamma q^2} \left(1 - \exp\left[-\Delta t / \tau_{\mathrm{mem}}\right]\right) \tag{S8}$$

$$\left\langle \left(a_{\mathrm{q}}(t+\Delta t) - a_{\mathrm{q}}(t)\right)^2 \right\rangle = \frac{2L}{L_{\mathrm{p}} q^2} \left(1 - \exp\left[-\Delta t / \tau_{\mathrm{wlc}}\right]\right) , \tag{S9}$$

where A is the projected area of the membrane patch and L is the end-to-end distance of the WLC.

Comparing measured characteristic decorrelation times τ to the expected behavior for membrane [4] and WLC [5],

$$\tau_{\mathrm{mem}} \sim \frac{4\eta}{\kappa q^3} \tag{S10}$$

$$\tau_{\mathrm{wlc}} \sim \frac{4\pi\eta}{L_{\mathrm{p}} k_{\mathrm{B}} T q^4} , \tag{S11}$$

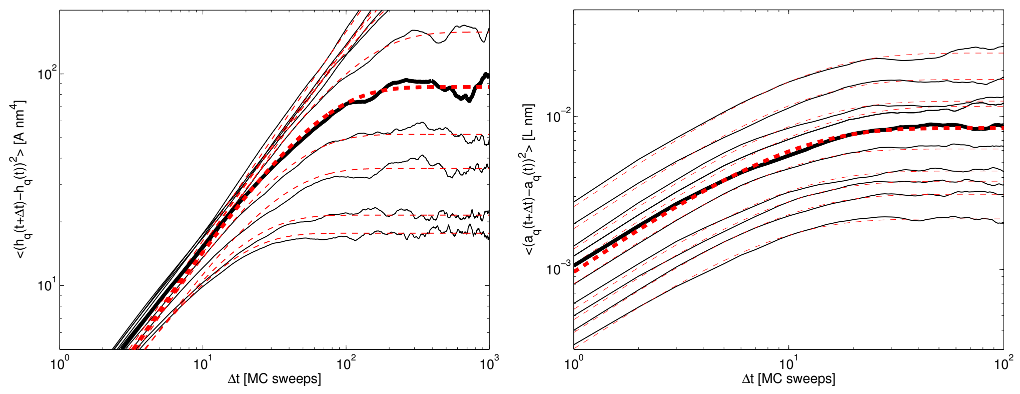

(a) (b)

FIG. S2: (a) Membrane normal mode decorrelation time Eq. (S8) for a quadratic membrane of linear size 200nm. (b) Worm like chain normal mode decorrelation time Eq. (S9) for a chain of 49 segments each of length $\delta = 2.7$nm.

reveals the simulation time scale in physical units. Here η is the viscosity of the solvent, i.e. water. Using characteristic modes with wavelength $\lambda_{\mathrm{mem}} \sim 30$nm for the membrane and wavelength $\lambda_{\mathrm{wlc}} \sim 45$nm in case of the worm-like chain, we obtain the same order of magnitude for the physical time scale of a single MC sweep in both systems, $\sim 10^{-10}$s.

2. Timescale of actin polymerization kinetics

The relaxation time of the relevant normal modes of membrane and WLC ($\tau_{\mathrm{mem}} \sim \tau_{\mathrm{wlc}} \sim 10^{-9}$s) is roughly seven orders of magnitude faster compared to the typical actin polymerization timescale that is realized in-vitro, $1/k_{\mathrm{on},0} \sim 10^{-2}$s, i.e. $\tau_{\mathrm{mem/wlc}} k_{\mathrm{on},0} \sim 10^{-7}$. Due to this large separation of timescales, we assume that both membrane and WLC equilibrate in between subsequent polymerization events and that correlations of their fluctuations can be neglected. For this reason, it is possible to speed up actin polymerization and depolymerization rates in simulation to the extent that membrane and filament equilibration is still satisfied after each event.

Stochastic filament (de-)polymerization events are realized by supplementing the combined membrane-WLC MC simulation, which has been gauged in time as described above, with a kinetic MC approach that draws a random reaction time and filament that is subject to the corresponding reaction [6]. While depolymerization can always take place, polymerization at the tip of a filament is only possible if sufficient space between the filament end

and any obstacle in its way is available at the time of the reaction. If polymerization is not possible due to such steric volume constraints of the membrane or other filaments, the reaction is rejected and a new reaction time and filament is selected randomly.

II. MASTER EQUATION APPROACH TO MEMBRANE INDUCED DYNAMIC FILAMENT BUNDLING

To quantify the dynamics of the filament bundling transition and calculate first passage time distributions to bundling, here we are developing a mathematical model based on a master equation for dynamic filament bending and growth, an important and limiting step to filament bundling. For this purpose, instead of the multi-filament–membrane system, we are exclusively considering a single filament growing against a rigid obstacle that responds linearly to deformations.

A. Filament polymerization as an anisotropic 1D random walk

As the waiting time between filament polymerization events – even in case of an unperturbed filament – is several orders of magnitude larger than the relaxation time of structural deformations of membrane and filament on the relevant length scale (cf. Sec. I B), at this point we are exclusively considering the effect of equilibrium fluctuations of a single filament growing against a harmonic rigid obstacle. Single filament polymerization against this obstacle can be understood as an anisotropic 1D random walk and modeled by the corresponding master equation,

$$\partial_t P(n,t) = k_{\mathrm{off}}\left[P(n+1,t) - P(n,t)\right] + k_{\mathrm{on}}(n-1)P(n-1,t) - k_{\mathrm{on}}(n)P(n,t)\,, \tag{S12}$$

where $P(n,t)$ is the probability that the filament consists of n monomers at time t, k_{off} and $k_{\mathrm{on}}(n)$ are the depolymerization and polymerization rates respectively. Polymerization rate is a function of filament length in terms of the number of monomer constituents, $L_{\mathrm{fil}} = n\delta_{\mathrm{fil}}$,

$$k_{\mathrm{on}}(n) = k_{\mathrm{on},0} P_{\mathrm{bend}}(n \to n+1)\,, \tag{S13}$$

with $k_{\mathrm{on},0}$, the free polymerization rate of an unperturbed filament and $P_{\mathrm{bend}}(n \to n+1)$, the equilibrium probability for a structural fluctuation that creates a sufficient gap of size

$\delta \geq \delta_{\text{fil}}$ between the obstacle and a filament of n monomers, such that the $(n+1)$th monomer fits in.

B. Equilibrium gap probability $P_{\text{bend}}(n \to n+1)$

To calculate $P_{\text{bend}}(n \to n+1)$, we are accounting for a single filament with fluctuating two dimensional curvature, $\kappa_{\text{fil}} \equiv 1/R$, which is constant along its contour of length $L_{\text{fil}} = n\delta_{\text{fil}}$. The filament grows against a rigid obstacle at initial angle θ and height L_0, measured relative to the filaments stationary base at $z_{\text{fil},0}$ as sketched in Fig. S3. In this approximation the worm-like chain Hamiltonian reduces to,

$$\mathcal{H}_{\text{wlc}} \simeq \frac{L_{\text{fil}}}{2} k_{\text{B}} T L_{\text{p}} \kappa_{\text{fil}}^2 \,, \tag{S14}$$

with filament persistence length L_{p} and thermal energy scale $k_{\text{B}}T$. The relative height of the obstacle L_0 fluctuates in the harmonic potential,

$$V_{\text{mem}} = \frac{k_{\text{mem}}}{2} \left(z_{\text{mem}} - z_{\text{mem},0}\right)^2 \,, \tag{S15}$$

where z_{mem} is the actual height of the obstacle and $z_{\text{mem},0}$ indicates the minimum of the harmonic potential. The spring constant k_{mem} is derived from the membrane's linear response to height deformations (cf. Sec. II C) and $L_0 = z_{\text{fil},0} - z_{\text{mem}}$, with the constant filament base height at $z_{\text{fil},0}$.

To calculate the probability for a gap of size $\delta \geq \delta_{\text{fil}}$ between the filament's growing end and the obstacle, we first have to express the gap size δ as a function of filament curvature, filament length, and obstacle distance,

$$\delta = \frac{z_{\text{fil},0} - z_{\text{mem}} - \frac{1}{\kappa_{\text{fil}}} \tan\left(\frac{L_{\text{fil}}\kappa_{\text{fil}}}{2}\right)}{\cos(L_{\text{fil}}\kappa_{\text{fil}}) - \frac{\sin(L_{\text{fil}}\kappa_{\text{fil}})}{\tan\theta}} - \frac{1-\cos(L_{\text{fil}}\kappa_{\text{fil}})}{\kappa_{\text{fil}} \sin(L_{\text{fil}}\kappa_{\text{fil}})} \,. \tag{S16}$$

Due to the constraint that the length of the filament is constant in between polymerization events, the position of the polymerizing end of the filament effectively fluctuates in one degree of freedom. Therefore, as a next step, we transform the 2D barbed end coordinates x and z to generalized coordinates, κ_{fil} and L_{fil}, which yields to lowest order in $L_{\text{fil}}\kappa_{\text{fil}}$,

$$x = \tfrac{1}{\kappa_{\text{fil}}} - \tfrac{1}{\kappa_{\text{fil}}} \cos\left(L_{\text{fil}}\kappa_{\text{fil}}\right) \simeq \frac{L_{\text{fil}}^2 \kappa_{\text{fil}}}{2} \tag{S17}$$

$$z = L_{\text{fil}} - \tfrac{1}{\kappa_{\text{fil}}} \sin\left(L_{\text{fil}}\kappa_{\text{fil}}\right) \simeq \frac{L_{\text{fil}}^3 \kappa_{\text{fil}}^2}{6} \,, \tag{S18}$$

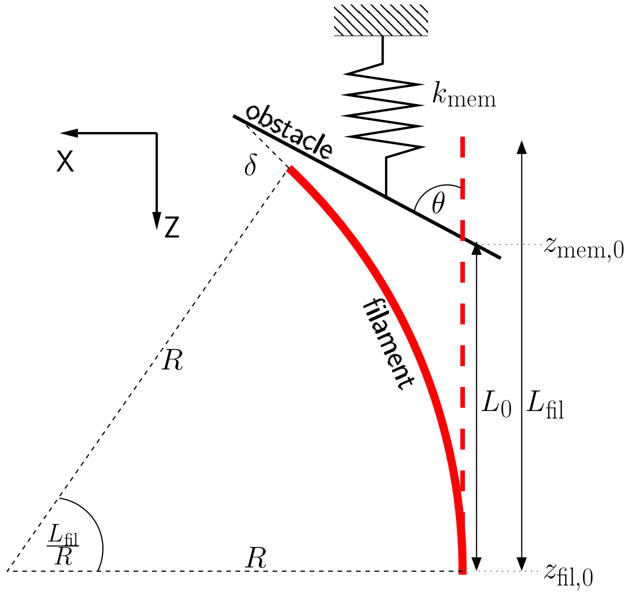

FIG. S3: Sketch of the simplified filament-obstacle system under consideration in the master equation model.

and due to the corresponding Jacobian determinant,

$$\mathrm{d}x\mathrm{d}z = \frac{1}{12} L_{\rm fil}^4 \kappa_{\rm fil}^2 \mathrm{d}L_{\rm fil} \mathrm{d}\kappa_{\rm fil}\,. \tag{S19}$$

For $L_{\rm fil} = \text{const.}$, this reduces to $\mathrm{d}x\mathrm{d}z \propto \kappa_{\rm fil}^2 \mathrm{d}\kappa_{\rm fil}\,.$

Combining this result with Eq. (S14) and Eq. (S15) subsequently leads to the gap probability,

$$P_{\rm bend}(n \to n{+}1) = \frac{\int\limits_{-\infty}^{z_{\rm fil,0}} \int\limits_{\kappa_{\rm fil}(\delta=\delta_{\rm fil})}^{\infty} \kappa_{\rm fil}^2 \exp\left[-\frac{L_{\rm P}}{2} L_{\rm fil} \kappa_{\rm fil}^2\right] \exp\left[-\frac{k_{\rm mem}}{2k_{\rm B}T}(z_{\rm mem} - z_{\rm mem,0})^2\right] \mathrm{d}\kappa_{\rm fil}\mathrm{d}z_{\rm mem}}{\int\limits_{-\infty}^{z_{\rm fil,0}} \int\limits_{\kappa_{\rm fil}(\delta=0)}^{\infty} \kappa_{\rm fil}^2 \exp\left[-\frac{L_{\rm P}}{2} L_{\rm fil} \kappa_{\rm fil}^2\right] \exp\left[-\frac{k_{\rm mem}}{2k_{\rm B}T}(z_{\rm mem} - z_{\rm mem,0})^2\right] \mathrm{d}\kappa_{\rm fil}\mathrm{d}z_{\rm mem}}\,, \tag{S20}$$

where the function $\kappa_{\rm fil}(\delta)$ is defined implicitly via Eq. (S16).

1. Numerical solution for $P_{\rm bend}(n \to n+1)$

To obtain the results of Fig. 4(b) in the main text, we have solved Eq. (S20) numerically, which results in Fig. S4(a).

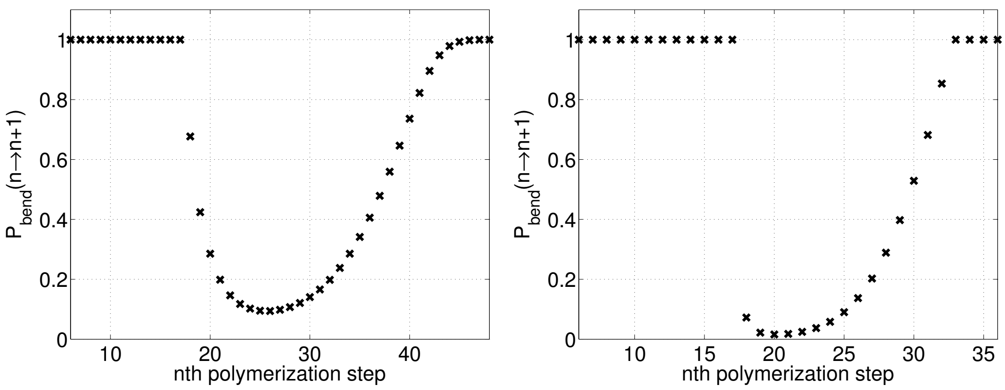

(a) (b)

FIG. S4: (a) Numerical solution for $P_{\rm bend}(n \to n+1)$, for representative parameters $z_{\rm fil,0} \simeq 46$nm, $z_{\rm mem,0} = 0$, $\theta = 45°$, $L_{\rm p} = 15\mu$m, and $k_{\rm mem} \simeq 0.1 k_{\rm B}T{\rm nm}^{-2}$. (b) $P_{\rm bend}(n \to n+1)$ for the same parameters as in (a) but in the limit of $k_{\rm mem} \to \infty$, i.e. as given by Eq. (S22). Note that in both situations, the gap probability is 1 before the filament end grows in reach of the obstacle. Once the dynamic bending transition occurred and the filament is sufficiently long, it polymerizes tangentially along the surface and $P_{\rm bend}$ approaches unity again.

2. $P_{\rm bend}(n \to n+1)$ in the limit of $k_{\rm mem} \to \infty$

As an illustrative alternative to the numerical solution of Eq. (S20) in Fig. S4(a), at this point we are also analyzing the limiting case of an infinitely stiff membrane obstacle, i.e. $k_{\rm mem} \to \infty$.

In this case Eq. (S20) reduces to,

$$P_{\rm bend}(n \to n+1) = \frac{\int\limits_{\kappa_{\rm fil}(\delta=\delta_{\rm fil})}^{\infty} \kappa_{\rm fil}^2 \exp\left[-\frac{L_{\rm p}}{2} L_{\rm fil} \kappa_{\rm fil}^2\right] {\rm d}\kappa_{\rm fil}}{\int\limits_{\kappa_{\rm fil}(\delta=0)}^{\infty} \kappa_{\rm fil}^2 \exp\left[-\frac{L_{\rm p}}{2} L_{\rm fil} \kappa_{\rm fil}^2\right] {\rm d}\kappa_{\rm fil}}\,, \tag{S21}$$

where we have used $\lim\limits_{k_{\rm mem}\to\infty} \sqrt{\frac{k_{\rm mem}}{2\pi k_{\rm B}T}} \exp\left[-\frac{k_{\rm mem}}{2k_{\rm B}T}(z_{\rm mem} - z_{\rm mem,0})^2\right] = \delta\left(z_{\rm mem} - z_{\rm mem,0}\right)$. Subsequently, Eq. (S21) yields,

$$P_{\rm bend}(n \to n+1) = \frac{\frac{\kappa_{\rm fil}(\delta=\delta_{\rm fil}) \exp\left[-\frac{L_{\rm p}}{2} L_{\rm fil} \kappa_{\rm fil}^2(\delta=\delta_{\rm fil})\right]}{L_{\rm p} L_{\rm fil}} + \frac{\sqrt{2\pi}{\rm erfc}\left[\sqrt{\frac{L_{\rm p}}{2} L_{\rm fil}}\kappa_{\rm fil}(\delta=\delta_{\rm fil})\right]}{2(L_{\rm p}L_{\rm fil})^{3/2}}}{\frac{\kappa_{\rm fil}(\delta=0) \exp\left[-\frac{L_{\rm p}}{2} L_{\rm fil} \kappa_{\rm fil}^2(\delta=0)\right]}{L_{\rm p} L_{\rm fil}} + \frac{\sqrt{2\pi}{\rm erfc}\left[\sqrt{\frac{L_{\rm p}}{2} L_{\rm fil}}\kappa_{\rm fil}(\delta=0)\right]}{2(L_{\rm p}L_{\rm fil})^{3/2}}}\,. \tag{S22}$$

Fig. S4(b) illustrates this result.

C. Linear response of the flat membrane patch

To estimate the spring stiffness $k_{\rm mem}$ of the simplified obstacle as a function of the membrane's elastic constants, bending rigidity κ and surface tension γ, we are calculating the linear response to a point force at the origin [7]. This eventually leads to the equation,

$$F = \frac{A^2 k_{\rm B}T}{\sum_{\mathbf{q}} \langle h_{\mathbf{q}}^2 \rangle} \langle h(\mathbf{x}=0) \rangle \ , \tag{S23}$$

which relates the membrane's height deformation $\langle h(\mathbf{x}=0)\rangle$ to the applied force F via the spring constant,

$$\hat{k}_{\rm mem} \equiv \frac{A^2 k_{\rm B}T}{\sum_{\mathbf{q}} \langle h_{\mathbf{q}}^2 \rangle} \ , \tag{S24}$$

with squared fluctuation amplitude,

$$\langle h_{\mathbf{q}}^2 \rangle = \frac{A k_{\rm B}T}{\kappa q^4 + \gamma q^2} \ . \tag{S25}$$

This leads to,

$$\sum_{\mathbf{q}} \langle h_{\mathbf{q}}^2 \rangle \simeq \int \frac{{\rm d}^2 q}{(2\pi)^2} \frac{k_{\rm B}T A^2}{\kappa q^4 + \gamma q^2} \tag{S26}$$

which can be either solved numerically or approximated in polar coordinates to yield,

$$\sum_{\mathbf{q}} \langle h_{\mathbf{q}}^2 \rangle \simeq \int_{q_{\rm min}}^{q_{\rm max}} \frac{{\rm d}q}{2\pi} \frac{k_{\rm B}T A^2}{\kappa q^3 + \gamma q} = \left[\frac{k_{\rm B}T A^2}{2\pi\gamma} \left(\ln[q] - \frac{1}{2}\ln[\kappa q^2 + \gamma] \right) \right]_{q_{\rm min}}^{q_{\rm max}} \tag{S27}$$

with low and high frequency cutoffs, $q_{\rm min} = 2\pi/L$ and $q_{\rm max} = \pi/\delta x$. Combining Eq. (S24) and Eq. (S27) results in the membrane's effective spring constant for vertical deformations,

$$\hat{k}_{\rm mem} \simeq \frac{2\pi\gamma}{\ln\left[\frac{L}{2\delta x}\right] - \frac{1}{2}\ln\left[\frac{\left(\frac{\pi}{\delta x}\right)^2 + \frac{\gamma}{\kappa}}{\left(\frac{2\pi}{L}\right)^2 + \frac{\gamma}{\kappa}}\right]} \ . \tag{S28}$$

The membrane's spring constant in the direction of the filament base (cf. Fig. S3) is thus given by,

$$k_{\rm mem} = \hat{k}_{\rm mem} \cos\theta \ . \tag{S29}$$

D. Mean first passage time

To obtain the first passage time distribution for the filament to reach a bend and growing state, where the polymerizing end is oriented tangentially to the membrane surface, we are

numerically iterating Eq. (S12) using a kinetic Monte Carlo approach [6]. Exemplary results are shown in Fig. 4(b) of the main text. The first moment of this distribution can be written as [8],

$$\mathrm{MFPT}(n_0) = \sum_{i=n_0}^{n_{\mathrm{final}}} \left(\prod_{l=2}^{i} \frac{k_{\mathrm{off}}}{k_{\mathrm{on}}(l)} \right) \sum_{j=1}^{i} \left(k_{\mathrm{on}}(j) \left(\prod_{l=2}^{j} \frac{k_{\mathrm{off}}}{k_{\mathrm{on}}(l)} \right) \right)^{-1}, \tag{S30}$$

where $n = 1$ is the initial and n_{final} is the bend filament state. These states are in good approximation perfectly reflecting and absorbing as $k_{\mathrm{on}}(n_{\mathrm{final}})/k_{\mathrm{off}} \simeq k_{\mathrm{on},0}/k_{\mathrm{off}} \sim 10^2$.

In Fig. 4(c) of the main text, we illustrate the mean first passage time to the bend state as a function of the important parameters L_0 and θ.

III. MOVIES

Two movies show simulations of filament ensemble growth against a freely fluctuating membrane (cf. main text Fig. 3(a) and `SI_movie_1`) and a membrane with one additional immobilized node (cf. main text Fig. 3(b) and `SI_movie_2`).

`SI_movie_3` corresponds to the filament-driven tube formation simulation discussed in Fig. 4(a) of the main text. `SI_movie_4` shows an example of the single filament bending transition under consideration in Fig. 4(b) of the main text.

`SI_movie_5` features the filament-driven tube formation simulation discussed in Fig. 5(a–c) of the main text.

---

[1] G. Gompper and D. M. Kroll, Journal of Physics: Condensed Matter **9**, 8795 (1997).

[2] S. Whitelam, Molecular Simulation **37**, 606 (2011).

[3] R. Shlomovitz, N. S. Gov, and A. Roux, New Journal of Physics **13**, 065008 (2011).

[4] M. C. Watson, Y. Peng, Y. Zheng, and F. L. H. Brown, The Journal of Chemical pPhysics **135**, 194701 (2011).

[5] C. P. Brangwynne, G. H. Koenderink, E. Barry, Z. Dogic, F. C. MacKintosh, and D. A. Weitz, Biophysical Journal **93**, 346 (2007).

[6] D. T. Gillespie, The Journal of Physical Chemistry **81**, 2340 (1977).

[7] J. Paulose, G. A. Vliegenthart, G. Gompper, and D. R. Nelson, Proceedings of the National Academy of Sciences of the United States of America **109**, 19551 (2012).

[8] C. Gardiner, *Stochastic Methods: A Handbook for the Natural and Social Sciences*, Springer Series in Synergetics (Springer, 2009), ISBN 9783540707127.

[9] Weisstein, Eric W. "Sphere Point Picking." From MathWorld–A Wolfram Web Resource. http://mathworld.wolfram.com/SpherePointPicking.html
